# Association between 24-Hour Urinary Cadmium and Pulmonary Function among Community-Exposed Men: The VA Normative Aging Study

**DOI:** 10.1289/ehp.11265

**Published:** 2008-05-14

**Authors:** Brad J. Lampe, Sung Kyun Park, Thomas Robins, Bhramar Mukherjee, Augusto A. Litonjua, Chitra Amarasiriwardena, Marc Weisskopf, David Sparrow, Howard Hu

**Affiliations:** 1 Department of Environmental Health Sciences, University of Michigan School of Public Health, Ann Arbor, Michigan, USA; 2 Department of Biostatistics, University of Michigan School of Public Health, Ann Arbor, Michigan, USA; 3 Channing Laboratory, Department of Medicine, Brigham and Women’s Hospital, Harvard Medical School, Boston, Massachusetts, USA; 4 VA Normative Aging Study, Veterans Affairs Boston Healthcare System, Boston, Massachusetts, USA; 5 Department of Medicine, Boston University School of Medicine, Boston, Massachusetts, USA

**Keywords:** cadmium, cigarette smoking, forced expiratory volume, forced vital capacity, pulmonary function

## Abstract

**Background:**

High levels of cadmium exposure are known to cause emphysema in occupationally exposed workers, but little has been reported to date on the association between chronic environmental cadmium exposure and pulmonary function.

**Objective:**

In this study we examined the association between pulmonary function and cadmium body burden in a subcohort of the Normative Aging Study, a community-based study of aging.

**Methods:**

We examined 96 men who had cadmium measured in single 24-hr urinary specimens collected in 1994–1995 and who had one to three tests of pulmonary function between 1994 and 2002 (a total of 222 observations). We used mixed-effect models to predict pulmonary function based on individual 24-hr urinary cadmium output, adjusted for age, height, time elapsed from the baseline, and smoking status. We assessed effect modification by smoking status.

**Results:**

Among all subjects, a single log-unit increase in baseline urinary cadmium was inversely associated with forced expiratory volume in 1 sec (FEV_1_) percent predicted [β = −7.56%; 95% confidence interval (CI) −13.59% to −1.53%]; forced vital capacity (FVC) percent predicted (β = −2.70%; 95% CI −7.39% to 1.99%), and FEV_1_/FVC ratio (β = −4.13%; 95% CI −7.61% to −0.66%). In models including an interaction between urinary cadmium and smoking status, there was a graded, statistically significant reduction in FEV_1_/FVC ratio across smoking status in association with urinary cadmium.

**Conclusions:**

This study suggests that chronic cadmium exposure is associated with reduced pulmonary function, and cigarette smoking modifies this association. These results should be interpreted with caution because the sample size is small, and further studies are needed to confirm our findings.

According to the World Health Organization (WHO 2007), 80 million people worldwide are afflicted with chronic obstructive pulmonary disease (COPD), and 3 million died from COPD in 2005. The WHO also predicts that COPD will be the fourth leading cause of death globally by 2030. Recent attention has focused on evaluating the relationship between pulmonary function and cadmium body burden and the possible role of cadmium in the development of pulmonary diseases such as COPD and emphysema.

Cadmium is a trace element that has no nutritive function in humans (Newman-Taylor 1998), and it is a probable lung carcinogen in humans according to the Agency for Toxic Substances and Disease Registry (ATSDR 1999) *Toxicological Profile for Cadmium*. The toxicity of cadmium in the lungs has been well documented in animal studies. For example, cadmium inhalation produces a pulmonary inflammatory response in mammals (Kirschvink et al. 2006), and daily doses of 1.6 mg/m^3^ cadmium aerosol over a 6-week period were associated with acute pulmonary damage and emphysema in rats (Hart 1986). Thus, pulmonary toxicity studies in animals support the hypothesis that reduced lung function among smokers may be partially attributable to cadmium in cigarette smoke. Primary nonoccupational sources of cadmium exposure within the general population include ingestion of contaminated food (Järup et al. 1998; Kido et al. 1992) and inhalation of cigarette smoke (Järup et al. 1998; Satarug and Moore 2004), and smokers have higher body burdens of cadmium than nonsmokers (Erzen and Kragelj 2006; Grasseschi et al. 2003; Järup et al. 1998; Mutti et al. 2006).

Although previous studies have examined the association between pulmonary function and cadmium exposure in animals (Hart 1986; Kirschvink et al. 2006; Lai and Diamond 1992) and in occupationally exposed cohorts (Cortona et al. 1992; Davison et al. 1988; Edling et al. 1986; Elinder 1986; Jakubowski et al. 2004; Lauwerys et al. 1979; Nordberg 2004; OSHA 1993; Sakurai et al. 1982; Smith et al. 1976; Stanescu et al. 1977), few studies have evaluated the association between cadmium exposure and pulmonary function in the general population.

A recent study evaluated the effects of cadmium exposure (measured by individual spot urinary concentrations) on pulmonary function among participants in the Third National Health Examination and Nutrition Examination Survey (NHANES III) (Mannino et al. 2004). Forced vital capacity (FVC), forced expiratory volume in 1 sec (FEV_1_), FEV_1_/FVC ratio, and a diagnosis of the Global Initiative on Obstructive Lung Disease (GOLD) stage II or higher COPD (defined as FEV_1_/FVC ratio < 0.7 and FEV_1_ < 80% predicted) were all inversely correlated with urinary cadmium concentrations in current and former smokers but not in nonsmokers (Mannino et al. 2004). The Mannino study was crucial in that it was the first to evaluate the relationship between cadmium body burden and pulmonary function using a quantifiable biomarker of exposure. Urinary cadmium is influenced by body burden and increases with age (Järup et al. 1998) and in proportion to the accumulated amount in the body (Elinder 1986), and urinary cadium is therefore generally recognized as an effective exposure surrogate.

The aim of this study was to examine the association between pulmonary function and cadmium body burden in a subcohort of the Normative Aging Study (NAS) while using multiple time points of pulmonary function measurements to control for subject-specific variation and to reduce residual confounding associated with subject age and time.

## Methods

### Study population

The NAS consists of a cohort of 2,280 healthy male volunteers from Boston, Massachusetts. Participants were mostly white, 21–80 years of age, and free of heart disease, hypertension, diabetes mellitus, cancer, peptic ulcer, gout, recurrent asthma, bronchitis, or sinusitus at the beginning of the study in 1961 (Bell et al. 1972). Since entry, participants have had regular medical screenings every 3–5 years consisting of a physical examination, blood and urine tests, and pulmonary function testing (Litonjua et al. 2005). In this study we analyzed a randomly selected subcohort of 96 NAS participants whose urinary cadmium was measured for a separate study assessing the relationship between urinary cadmium and dietary intake. All 96 participants had a 24-hr urinary specimen (collected between March 1994 and November 1995) and met the following criteria: *a*) subject’s 24-hr urine volume collected was ≥ 3,000 mL, *b*) no urine was spilled during collection, *c*) subject’s creatinine clearance was ≥ 50 in order to exclude subjects with poor renal function, and *d*) the subject had complete information on dietary intake.

Participants visited the study center in the morning after abstaining from smoking. Physical examinations included measurement of height, weight, and lung function (FVC, FEV_1_) testing. We assessed smoking status and intensity (pack-years of cigarettes) using the American Thoracic Society Division of Lung Diseases 1978 questionnaire (Ferris 1978). All participants provided written informed consent. This study was reviewed and approved by the institutional review boards of all participating institutions.

### Pulmonary function testing

All pulmonary tests were performed according to American Thoracic Society standard methodology (ATS 1987). Each subject made up to eight FVC maneuvers to obtain three acceptable curves according to predefined criteria, and standard methods were used to obtain FVC and FEV_1_ (Litonjua et al. 2005). We used the largest value of the three maneuvers for either measure (not necessarily from the same curve) for this analysis, and all values were corrected to body temperature and pressure saturated with water vapor (Litonjua et al. 2005). Pulmonary function data including FEV_1_ and FVC measurements between 1994 and 2002 were available for all 96 subjects, for a total of 222 observations (3 measurements for 46 subjects; 2 measurements for 34 subjects; 1 measurement for 16 subjects). The mean duration between successive pulmonary tests was approximately 3.1 years.

We calculated FEV_1_ and FVC as the percentage of predicted values using the prediction equations from NHANES III (Hankinson et al. 1999). For white males ≥ 20 year of age, the following equations are suggested:


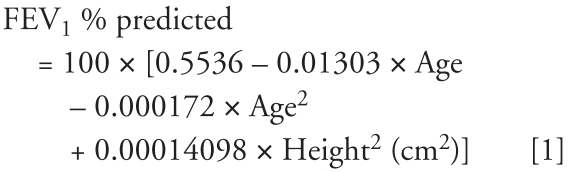



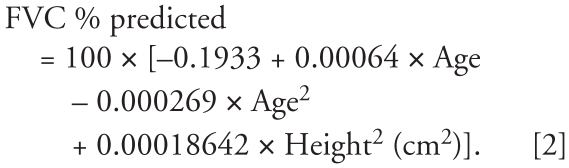


### Urinary cadmium analysis

For each subject, urinary cadmium samples and pulmonary function tests were given on the same date during between March 1994 and November 1995. All urine samples were handled in a class-100 clean hood. All glassware and plastics were cleaned by soaking in 10% nitric acid (HNO_3_) for 24 hr and rinsing several times with deionized water. Reagents used in this study were HNO_3_ (Optima; Seastar Chemical Co., Pittsburgh, PA); National Institute of Standard and Technology Standard Reference Material (NIST SRM) 1643d (Gaithersburg, MD); enriched isotope spike ^113^Cd (Oak Ridge National Laboratory, Oak Ridge, TN). We mixed 5 mL of urine sample with 1 mL of isotope dilution spike (40 ppb ^113^Cd-enriched isotope dilution spike) and 20 μL of chelating resin [10% SPR-IDA (suspended particulate reagent–iminodiacetate), 10 μL; CETAC Technologies (Omaha, NE)] in a plastic centrifuge tube. After adjusting the pH of the solution to 8, samples were centrifuged at 5,000 rpm for 20 min. Supernatant was discarded and resin was washed with deionized water adjusted to a pH of 8. Then 2 mL of 5% HNO_3_ was added to the precipitate, and it was centrifuged at 5,000 rpm for 20 min. The cadmium-containing supernatant was then removed and analyzed by inductively coupled plasma mass spectrometry (Sciex Elan 5000; PerkinElmer, Norwalk, CT), with standard instrument operating and data collection parameters, using the isotope dilution procedure. Quality control and quality assurance procedures included analyses of procedural blanks, duplicate analysis, spiked samples, and analysis of NIST SRM 2670 (toxic metals in freeze-dried urine), and NIST SRM 1643d (trace elements in water) to monitor the accuracy and recovery rates of the procedure for each analytic batch. We report results as the average of five replicate measurements. Recoveries of these quality control standards were between 90% and 110% in our laboratory. The calculated detection limit for Cd analysis by this procedure was 0.02 ng/mL of sample (urinary Cd for all 96 samples > 0.02 ng/mL). Urinary cadmium measurements used in this study were adjusted for individual 24-hr urine output.

### Statistical analysis

Statistical analysis was carried out using SAS (version 9.1; SAS Institute Inc., Cary, NC), and the primary outcome measures were FEV_1_ percent predicted, FVC percent predicted, and FEV_1_/FVC ratio. We log-transformed 24-hr urinary cadmium outputs (U-Cd) to normalize the distribution. Because our pulmonary function outcomes were measured repeatedly, we fit a mixed-effects models using the PROC MIXED procedure in SAS to deal with lack of independence of observations. For FEV_1_/FVC ratio, we considered the following covariates: baseline age (years), baseline height (cm), time elapsed from the baseline (years), and smoking status [never smokers, former smokers with low pack-years (≤ 30), former smokers with high pack-years (> 30), and current smokers], where baseline is defined as the time point of urinary cadmium and pulmonary function measurements during the period between March 1994 and November 1995. For FEV_1_ percent predicted and FVC percent predicted, baseline age and height were not included because the percentage of predicted value already accounts for age and height. Because smoking status is the only time-dependent variable, the interaction between time and smoking status was included. We also included an interaction between time and urinary cadmium in the model to test whether the association between urinary cadmium and lung functions changes over time. A random slope for the time elapsed from the baseline was initially considered to account for subject-specific variability of lung function over time, but a random intercept-only model was preferred based on the likelihood ratio test comparing the two models. The following equation describes the structure of our fitted models:


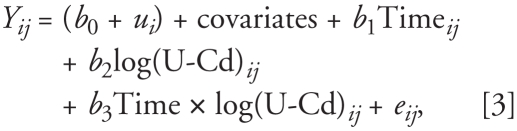


where *Y_ij_* is the lung function in subject *i* at time *j*, *b*_0_ is the overall intercept, *u_i_* is the separate random intercept for subject *i*, *b*_1_ is the slope representing the overall effect of time, *b*_2_ is the slope representing the overall effect of log-transformed U-Cd, and *b*_3_ is the slope for the interaction between time and U-Cd. As a sensitivity analysis, we also re-ran the same mixed models using raw lung function measures (FEV_1_ and FVC, in liters) further adjusted for baseline age and height.

To evaluate effect modification by smoking status, we introduced an interaction term between categories of smoking status and log-transformed U-Cd, and tests for linear trend of U-Cd across the four categories of smoking status were computed.

## Results

Geometric mean 24-hr urinary cadmium output and mean pulmonary function measurements at baseline stratified by age, height, and smoking status are presented in Table 1. Current smokers had a higher geometric mean U-Cd and a lower mean FEV_1_/FVC ratio than both former smokers and never smokers, and former smokers who reported smoking > 30 pack-years had a higher mean U-Cd and a lower mean FEV_1_/FVC ratio than both former smokers reporting ≤ 30 pack-years and never smokers (*p*-values for trend < 0.05).

Table 2 shows the adjusted regression results of the main effects models of U-Cd with FEV_1_ percent predicted, FVC percent predicted, or FEV_1_/FVC ratio as the primary outcome measurement. Among all subjects, a single log-unit increase in U-Cd was inversely associated with FEV_1_ percent predicted [β = −7.56%; 95% confidence interval (CI), −13.59 to −1.53%], FVC percent predicted (β = −2.70%; 95% CI, −7.39 to 1.99%), and FEV_1_/FVC ratio (β = −4.13%; 95% CI, −7.61 to −0.66%) at baseline after adjusting for potential confounders. No statistically significant interactions between log U-Cd and time were found. The main-effects models also showed that current smokers had borderline significantly lower FEV_1_ percent predicted (β = −10.70%; 95% CI, −21.40 to 0.00) compared with never smokers, and there was a gradual reduction in FEV_1_ percent predicted across categories of smoking status (*p*-value for trend = 0.07). Additionally, there was a marginally significant increasing trend in FEV_1_/FVC ratio decline over time across categories of smoking status (*p*-value for trend = 0.08). The models using raw FEV_1_ and FVC measured in liters additionally adjusted for baseline age and height gave similar results to those found using the percentage of predicted values (data not shown).

**Figure 1:**
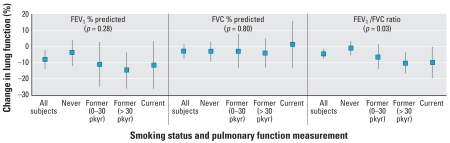
Estimated change (95% confidence interval) in FEV_1_ percent predicted, FVC percent predicted, and FEV_1_/FVC ratio for every log-unit increase in 24-hr urinary cadmium output by smoking status. pkyr, pack-years. Each model was adjusted for smoking status, time elapsed from the baseline, and the interaction between smoking status and time. FEV_1_/FVC ratio model was also adjusted for baseline age and height. The *p*-values above each pulmonary function are for tests for trend (*n* = 96 subjects, 222 pulmonary function observations, with a maximum of 3 pulmonary function observations per subject).

We assessed whether smoking status modified the effect of U-Cd on lung function (Figure 1). Because the interaction between log U-Cd and time was not statistically significant, this term was excluded when we tested the interaction between U-Cd and smoking status. A log-unit increase in urinary cadmium output was associated with −0.90% (95% CI, −5.37 to 3.58%), −6.22% (95% CI, −13.87 to 1.44%), −10.06% (95% CI, −16.49 to −3.64%), and −9.97% (95% CI, −19.60 to −0.34%) in FEV _1_ /FVC ratio among never smoker, former smokers with low pack-years, former smokers with high pack-years, and current smokers, respectively.For FEV_1_/FVC ratio, there was a trend of increasing magnitude of effect of U-Cd on pulmonary function with increasing smoking status category (*p*-value for trend = 0.03). The same trend was seen for FEV_1_ percent predicted, with the largest effect of U-Cd among former smokers with greater than 30 pack-years. The effect of U-Cd among current smokers was comparable but slightly weaker (*p*-value for trend = 0.28). In contrast, U-Cd was not significantly associated with FVC percent predicted, and smoking status did not seem to modify the association between U-Cd and FVC in our cohort.

## Discussion

In this study cohort, U-Cd was significantly associated with lower FEV_1_ as the percentage of predicted values and FEV_1_/FVC ratio after adjustment for potential confounders. We also observed a significant trend of increased effect of U-Cd on pulmonary function, especially FEV_1_/FVC ratio, with increasing smoking status category. However, U-Cd was not associated with the rate of change in lung function over the period of observation.

This finding is consistent with a cross-sectional study on urinary cadmium body burden and pulmonary function using data from NHANES III (Mannino et al. 2004). Other studies that have examined this association in human populations have been primarily concerned with occupational exposure, and the results are somewhat inconsistent. For example, inverse associations between occupational exposure to cadmium and pulmonary function have been suggested in several studies (Davison et al. 1988; Edling et al. 1986; Jakubowski et al. 2004; Nordberg 2004; Sakurai et al. 1982; Smith et al. 1976), but the effect estimates of cadmium exposure on pulmonary function and the methods used among these studies varied widely, making it difficult to confirm a definitive dose–response relationship. In addition, other occupational studies of cadmium exposure and pulmonary function have found no such association (Cortona et al. 1992; Lauwerys et al. 1979; Stanescu et al. 1977), although some of these latter studies did not control for smoking status among their study groups, raising methodological issues (Elinder 1986). Despite these uncertainties, it remains generally accepted that chronic exposure to cadmium fumes in the workplace is a risk factor for reduced pulmonary function (Elinder 1986; Occupational Safety and Health Administration 1993), supporting the hypothesis that environmental cadmium exposure may be a significant contributor to reduced pulmonary function among smokers.

Animal studies also support a link between cadmium exposure and reduced pulmonary function. For example, rats exposed to cadmium aerosol at a concentration of 1.6 mg/m^3^ for 2 weeks had increased leukocyte concentrations and alveolar thickening in the lung (Hart 1986). The administration of three intratracheal injections of cadmium chloride over a 5-day period was associated with decreased lung capacity and increased alveolar wall thickness in rats (Lai and Diamond 1992). Furthermore, repeated 1-hr exposures to 0.1% cadmium chloride for 3 weeks led to higher bronchoalveolar lavage fluid concentrations of macrophages and neutrophils in rats, as well as to decreased levels of glutathione during the first week of exposure (Kirschvink et al. 2006). These results suggest that cadmium exposure can lead to an acute inflammatory reaction accompanied by a buildup of free radicals (evidenced by decreased glutathione levels), which can consequently lead to lung inflammation (Kirschvink et al. 2006). Thus, in animals cadmium is associated with physiological changes indicative of reduced pulmonary function.

We observed a dose-dependent relationship between urinary cadmium and FEV_1_/FVC ratio with increasing smoking status category. It has previously been shown that smokers have higher body burdens of cadmium compared with nonsmokers, which may explain why the strength of the association between urinary cadmium and pulmonary function depends on smoking intensity (Lewis et al. 1972). However, because urinary cadmium was associated with pulmonary function even after adjusting for smoking status, smoking factors alone cannot explain the association. The cadmium in cigarette smoke may exert a toxic effect that is independent of the combined effects of the non-cadmium elements of cigarette smoke.

It was also surprising that we did not find a strong association between 24-hr U-Cd and FVC. Although the NHANES III study did find an inverse association between creatinine-adjusted urinary cadmium and FVC (Mannino et al. 2004), the association was weaker than that between urinary cadmium and FEV_1_. It is possible that, due to sample size (*n* = 96), we did not have the statistical power to detect the association between urinary cadmium and FVC.

We also did not find any suggestion of an effect among nonsmokers. One potential explanation is that cadmium’s effect on lung function requires airborne exposure and pulmonary deposition, a possibility also noted by Hendrick (2004) when commenting on the same phenomenon in Mannino et al.’s (2004) study of cadmium and lung function in NHANES III data. An alternative explanation, as Hendrick points out, is that cadmium body burden may simply be a passive marker of exposure to cigarette smoke and unrelated to the decrements in lung function seen among current and former smokers in our study. However, because there was a significant effect of cadmium on lung function even after controlling for smoking status, the results are consistent with an independent effect of cadmium. In addition, although smoking would clearly be the dominant source of airborne cadmium exposure to any given individual, it is well known that cadmium content varies widely among cigarettes (depending on cadmium levels in soil used to grow tobacco, methods of processing, and the like) (Yue 1992), which may explain why we would see an effect of cadmium on lung function that is independent from an effect of smoking. Unfortunately, our study does not answer the question of whether other metals that occur in cigarette smoke, such as lead and aluminum, may enhance the effect of cadmium on lung function. Mutti et al. (2006) demonstrated that these elements are abundant along with cadmium in the exhaled breath condensate of current smokers. Cadmium and lead both have mechanisms of toxicity that could result in the physiological changes that cause reduced lung function: Cadmium inhibits the production of connective tissue in the lungs, and lead may cause glutathione depletion in the lungs (Mutti et al. 2006). It cannot be ruled out, therefore, that these two effects (as well as others) may be acting in tandem to result in the decrements in lung function seen among the subjects in this study.

In the NHANES III study, urinary cadmium concentrations were adjusted for creatinine concentration (Mannino et al. 2004). In this study, creatinine-adjusted urine concentrations were not used because we had more complete data on 24-hr urinary output and thus we adjusted urinary cadmium for total urinary output instead. Adjustment for urinary creatinine is required for spot samples because of concentration dilution of urine, but creatinine adjustment may not be necessary for 24-hr urine samples (Berlin et al. 1985). The use of certain methods for the adjustment of urinary cadmium output for hydration status may also influence the effect of smoking status on urinary cadmium and pulmonary function.

In this study, most subjects had multiple time points of pulmonary function data, which allowed us to reduce the effect of within-subject variability in pulmonary function over time. It also helped us to explore time-dependent behavior of smoking status in the model and eliminate any trend effects due to time that are not accounted for by the other variables. However, there were some limitations to this study. First, our study cohort was small (*n* = 96) compared with similar analyses drawn from large-scale cohort studies such as NAS and NHANES III, and thus our statistical power is comparatively low. Second, although we had repeated pulmonary function data, we only had one valid time point (1994–1995) of urinary cadmium data per subject. Thus, we had to assume that the 24-hr urinary cadmium outputs from 1994–1995 were representative of the actual 24-hr urinary cadmium outputs for the entire time (1994–2002) during which pulmonary data were collected. This might be a reason that we did not find a significant association of urinary cadmium with the rate of change in lung function. In addition, we had only at most three measurements of lung function per subject, so we may not have the power to detect such an association. Third, we cannot say whether high urinary cadmium output physiologically precedes decrements in pulmonary function over time because there was not a long enough follow-up period between urinary cadmium measurement and pulmonary testing to make that kind of causal inference. Fourth, we lacked data on urinary cotinine levels and therefore we did not have an objective measurement of smoking intensity. Finally, we lacked precise data on the duration of time since quitting among the cohort of former smokers in our study, so we were unable to take into consideration that former smokers may differ in susceptibility to the effects of cadmium exposure depending on how long they have abstained from smoking.

In conclusion, we found significant evidence that there exists an inverse association between baseline cadmium body burden and pulmonary function, but the association does not change over time. Our results also suggest that cigarette smoking modifies this association. However, the present study should be interpreted with caution because of the small sample size; further studies with a larger sample size as well as a longitudinal design are needed to confirm our findings. The availability of more complete information on cigarette smoking, such as plasma cotinine levels, may also help elucidate the effect of smoking status on the association between cadmium exposure and pulmonary function.

## Figures and Tables

**Table 1:** Frequency distributions, geometric mean 24-hr urinary cadmium outputs, and mean pulmonary function measurements at baseline stratified by age, height, and smoking status (*n* = 96).

Variable	No. (%)	24-hr U-Cd (ng)^a^	FEV_1_ (% predicted)^b^	FVC (% predicted)^b^	FEV_1_/FVC ratio (%)^b^
Baseline age (years)					
< 65	36 (37.5)	615.5 (1.9)	95.3 (14.8)	91.9 (12.3)	78.3 (7.2)
65–70	26 (27.1)	566.9 (2.2)	91.2 (17.4)	90.8 (13.4)	74.2 (8.2)
> 70	34 (35.4)	577.2 (1.8)	92.3 (20.9)	87.0 (12.8)	76.4 (12.2)
*p* for trend		0.68	0.48	0.11	0.39
Baseline height (cm)					
< 170	28 (29.2)	573.1 (2.0)	98.4 (14.4)	91.9 (10.3)	78.9 (8.6)
170–180	26 (27.1)	585.9 (2.1)	91.3 (18.6)	90.1 (13.7)	72.5 (11.3)
> 180	42 (43.8)	600.4 (1.8)	89.6 (19.2)	85.0 (13.6)	77.4 (8.4)
*p* for trend		0.93	0.08	0.13	0.43
Smoking status					
Never	35 (36.5)	394.3 (2.0)	97.5 (16.8)	91.8 (12.5)	78.6 (8.9)
Former (0–30 pack-years)	32 (33.3)	621.5 (1.5)	97.7 (15.3)	92.8 (11.2)	77.9 (8.4)
Former (> 30 pack-years)	18 (18.8)	766.0 (1.8)	86.3 (18.3)	83.8 (12.6)	75.3 (11.8)
Current	11 (11.5)	1165.0 (1.6)	77.5 (15.7)	85.3 (15.7)	67.8 (6.2)
*p* for trend		< 0.001	< 0.001	0.027	0.002

a Geometric mean (geometric SD).

b Arithmetic mean (SD).

**Table 2:** Adjusted estimates (95% confidence intervals) for FEV_1_ percent predicted, FVC percent predicted, and FEV_1_/FVC ratio from the main effects models of urinary cadmium a.

Characteristic	FEV_1_ (% predicted)	FVC (% predicted)	FEV_1_/FVC ratio (%)
Intercept	142.2 (105.7 to 178.6)	107.6 (79.28 to 136.0)	130.0 (67.50 to 192.6)
Baseline age (years)	—	—	−0.03 (−0.33 to 0.26)
Baseline height (cm)	—	—	−0.14 (−0.45 to 0.16)
Smoking status			
Never	Referent	Referent	Referent
Former (0–30 pack-years)	3.63 (−4.77 to 12.04)	2.01 (−4.49 to 8.52)	1.54 (−3.30 to 6.39)
Former (> 30 pack-years)	−6.83 (−16.58 to 2.92)	−5.33 (−13.03 to 2.37)	−1.30 (−7.03 to 4.42)
Current	−10.70 (−21.40 to 0.00)	−7.00 (−16.07 to 2.06)	−3.17 (−9.64 to 3.31)
Time elapsed from the baseline (years)	1.00 (−1.90 to 3.90)	−0.12 (−3.74 to 3.51)	0.94 (−1.29 to 3.17)
Smoking × time			
Never	Referent	Referent	Referent
Former (0–30 pack-years)	0.49 (−0.19 to 1.17)	0.29 (−0.56 to 1.14)	0.06 (−0.46 to 0.58)
Former (> 30 pack-years)	−0.55 (−1.46 to 0.36)	0.15 (−0.98 to 1.28)	−0.47 (−1.16 to 0.23)
Current	−0.32 (−1.76 to 1.11)	1.15 (−0.62 to 2.92)	−1.07 (−2.16 to 0.03)
LogU-Cd (ng)	−7.56 (−13.59 to −1.53)	−2.70 (−7.39 to 1.99)	−4.13 (−7.61 to −0.66)
LogU-Cd × time	−0.19 (−0.67 to 0.29)	−0.10 (−0.70 to 0.50)	−0.11 (−0.47 to 0.26)

a Sample size = 96 subjects, 222 pulmonary function observations, with a maximum of 3 pulmonary function observations per subject.

## References

[b1-ehp-116-1226] American Thoracic Society (1987). Standardization of spirometry—1987 update. Statement of the American Thoracic Society. Am Rev Respir Dis.

[b2-ehp-116-1226] ATSDR (1999). Toxicological Profile for Cadmium. Atlanta, GA: Agency for Toxic Substances and Disease Registry.

[b3-ehp-116-1226] Bell B, Rose C, Damon A (1972). The Normative Aging Study: an interdisciplinary and longitudinal study of health and aging. Aging Human Dev.

[b4-ehp-116-1226] Berlin A, Alessio L, Sesana G, Dell’Orto A, Ghezzi I (1985). Problems concerning the usefulness of adjustment of urinary cadmium for creatinine and specific gravity. Int Arch Occup Environ Health.

[b5-ehp-116-1226] Cortona G, Apostoli P, Toffoletto F, Baldasseroni A, Ghezzi I, Goggi E (1992). Occupational exposure to cadmium and lung function. IARC Sci Publ.

[b6-ehp-116-1226] Davison AG, Fayers PM, Taylor AJ, Venables KM, Darbyshire J, Pickering CA (1988). Cadmium fume inhalation and emphysema. Lancet.

[b7-ehp-116-1226] Edling C, Elinder CG, Randma E (1986). Lung function in workers using cadmium containing solders. Br J Ind Med.

[b8-ehp-116-1226] Elinder C, Friberg L, Elinder C, Kjellstrom T, Nordberg GF (1986). Respiratory effects. Cadmium and Health: A Toxicological and Epidemiological Appraisal: Effects and Response.

[b9-ehp-116-1226] Erzen I, Kragelj LZ (2006). Cadmium concentrations in blood in a group of male recruits in Slovenia related to smoking habits. Bull Environ Contam Toxicol.

[b10-ehp-116-1226] Ferris BG (1978). Epidemiology Standardization Project (American Thoracic Society). Am Rev Respir Dis.

[b11-ehp-116-1226] Grasseschi RM, Ramaswamy RB, Levine DJ, Klaassen CD, Wesselius LJ (2003). Cadmium accumulation and detoxification by alveolar macrophages of cigarette smokers. Chest.

[b12-ehp-116-1226] Hankinson JL, Odencrantz JR, Fedan KB (1999). Spirometric reference values from a sample of the general U.S. population. Am J Respir Crit Care Med.

[b13-ehp-116-1226] Hart BA (1986). Cellular and biochemical response of the rat lung to repeated inhalation of cadmium. Toxicol Appl Pharmacol.

[b14-ehp-116-1226] Hendrick DJ (2004). Smoking, cadmium, and emphysema. Thorax.

[b15-ehp-116-1226] Jakubowski M, Abramowska-Guzik A, Szymczak W, Trzcinka-Ochocka M (2004). Influence of long-term occupational exposure to cadmium on lung function tests results. Int J Occup Med Environ Health.

[b16-ehp-116-1226] Järup L, Berglund M, Elinder CG, Nordberg G, Vahter M (1998). Health effects of cadmium exposure—a review of the literature and a risk estimate. Scand J Work Environ Health.

[b17-ehp-116-1226] Kido T, Nogawa K, Ohmichi M, Honda R, Tsuritani I, Ishizaki M (1992). Significance of urinary cadmium concentration in a Japanese population environmentally exposed to cadmium. Arch Environ Health.

[b18-ehp-116-1226] Kirschvink N, Martin N, Fievez L, Smith N, Marlin D, Gustin P (2006). Airway inflammation in cadmium-exposed rats is associated with pulmonary oxidative stress and emphysema. Free Radic Res.

[b19-ehp-116-1226] Lai YL, Diamond L (1992). Cigarette smoke exposure does not prevent cadmium-induced alterations in rat lungs. J Toxicol Environ Health.

[b20-ehp-116-1226] Lauwerys RR, Roels HA, Buchet JP, Bernard A, Stanescu D (1979). Investigations on the lung and kidney function in workers exposed to cadmium. Environ Health Perspect.

[b21-ehp-116-1226] Lewis GP, Coughlin LL, Jusko WJ, Hartz S (1972). Contribution of cigarette smoking to cadmium accumulation in man. Lancet.

[b22-ehp-116-1226] Litonjua AA, Lazarus R, Sparrow D, Demolles D, Weiss ST (2005). Lung function in type 2 diabetes: the Normative Aging Study. Respir Med.

[b23-ehp-116-1226] Mannino DM, Holguin F, Greves HM, Savage-Brown A, Stock AL, Jones RL (2004). Urinary cadmium levels predict lower lung function in current and former smokers: data from the Third National Health and Nutrition Examination Survey. Thorax.

[b24-ehp-116-1226] Mutti A, Corradi M, Goldoni M, Vettori MV, Bernard A, Apostoli P (2006). Exhaled metallic elements and serum pneumoproteins in asymptomatic smokers and patients with COPD or asthma. Chest.

[b25-ehp-116-1226] Newman-Taylor AJ, Rom WN (1998). Cadmium. Environmental and Occupational Medicine.

[b26-ehp-116-1226] Nordberg GF (2004). Cadmium and health in the 21st century—historical remarks and trends for the future. Biometals.

[b27-ehp-116-1226] Occupational Safety and Health Administration (1993). Occupational exposure to cadmium. Fed Reg.

[b28-ehp-116-1226] Sakurai H, Omae K, Toyama T, Higashi T, Nakadate T (1982). Cross-sectional study of pulmonary function in cadmium alloy workers. Scand J Work Environ Health.

[b29-ehp-116-1226] Satarug S, Moore MR (2004). Adverse health effects of chronic exposure to low-level cadmium in foodstuffs and cigarette smoke. Environ Health Perspect.

[b30-ehp-116-1226] Smith TJ, Petty TL, Reading JC, Lakshminarayan S (1976). Pulmonary effects of chronic exposure to airborne cadmium. Am Rev Respir Dis.

[b31-ehp-116-1226] Stanescu D, Veriter C, Frans A, Goncette L, Roels H, Lauwerys R (1977). Effects on lung of chronic occupational exposure to cadmium. Scand J Respir Dis.

[b32-ehp-116-1226] WHO (2007). Chronic Obstructive Pulmonary Diseases.

[b33-ehp-116-1226] Yue L (1992). Cadmium in tobacco. Biomed Environ Sci.

